# Investigation of the Mechanism of Traditional Chinese Medicines in Angiogenesis through Network Pharmacology and Data Mining

**DOI:** 10.1155/2021/5539970

**Published:** 2021-04-28

**Authors:** Wingyan Yun, Wenchao Dan, Jinlei Liu, Xinyuan Guo, Min Li, Qingyong He

**Affiliations:** ^1^Guang'anmen Hospital, China Academy of Chinese Medical Sciences, Beijing 100053, China; ^2^Graduate School of Beijing University of Chinese Medicine, Beijing 100029, China; ^3^Cancer Hospital Chinese Academy of Medical Sciences, Beijing 100021, China

## Abstract

Although traditional Chinese medicine is effective and safe for the treatment of angiogenesis, the *in vivo* intervention mechanism is diverse, complex, and largely unknown. Therefore, we aimed to explore the active ingredients of traditional Chinese medicine and their mechanisms of action against angiogenesis. Data on angiogenesis-related targets were collected from GeneCards, Therapeutic Target Database, Online Mendelian Inheritance in Man, DrugBank, and DisGeNET. These were matched to related molecular compounds and ingredients in the traditional Chinese medicine system pharmacology platform. The data were integrated and based on the condition of degree > 1, and relevant literature, target-compound, compound-medicine, and target-compound-medicine networks were constructed using Cytoscape. Molecular docking was used to predict the predominant binding combination of core targets and components. We obtained 79 targets for angiogenesis; 41 targets were matched to 3839 compounds, of which 110 compounds were selected owing to their high correlation with angiogenesis. Fifty-five combinations in the network were obtained by molecular docking, among which PTGS2-astragalin (−9.18 kcal/mol), KDR-astragalin (−7.94 kcal/mol), PTGS2-quercetin (−7.41 kcal/mol), and PTGS2-myricetin (−7.21 kcal/mol) were top. These results indicated that the selected potential core compounds have good binding activity with the core targets. Eighty new combinations were obtained from the network, and the top combinations based on affinity were KDR-beta-carotene (−10.13 kcal/mol), MMP9-beta-sitosterol (−8.04 kcal/mol), MMP9-astragalin (−7.82 kcal/mol), and MMP9-diosgenin (−7.51 kcal/mol). The core targets included PTGS2, KDR, VEGFA, and MMP9. The essential components identified were astragalin, kaempferol, myricetin, quercetin, and *β*-sitosterol. The crucial Chinese medicines identified included Polygoni Cuspidati Rhizoma et Radix, *Morus alba* Root Bark, and Forsythiae Fructus. By systematically analysing the ingredients of traditional Chinese medicine and their targets, it is possible to determine their potential mechanisms of action against pathological angiogenesis. Our study provides a basis for further research and the development of new therapeutics for angiogenesis.

## 1. Introduction

John Hunter provided the first recorded scientific insights into angiogenesis and coined the term angiogenesis in 1787 [1]. Folkman [2–6] proposed the role of angiogenesis in tumour growth in 1971. He hypothesised that tumour growth depends on angiogenesis to increase blood supply and proposed stopping the blood supply from inhibiting tumour growth, which subsequently initiated the field of research on the relationship between angiogenesis and diseases. Angiogenesis is the process of capillary sprouting from preexisting vasculature, and it is highly induced by hypoxia and other biological processes [5, 7]. The mechanisms underlying angiogenesis can be divided into two types. The first is sprouting angiogenesis, wherein vascular endothelial cell growth factor (VEGF) stimulates tip cells in the original blood vessel network to induce vascular sprouting [8, 9]. The second is intussusceptive angiogenesis, which proceeds through transluminal tissue pillar formation and subsequent vascular splitting to the expansion and remodelling of microvascular networks [9–11].

Under normal circumstances, angiogenesis is a balance between inhibiting and growth factors. If the functions of either the inhibiting or growth factors are abnormal, it presents as overgrowth, defect, or malformation. Angiogenesis is essential for the growth and development of tumour cells. Under hypoxic conditions, tumours stimulate neovascularisation via the expression of growth factors such as VEGF [12]. Thus, exploring the inhibition of angiogenesis for the treatment of tumours has gained increased attention. However, some studies have shown that using antiangiogenic agents can induce potential resistance mechanisms such as autophagy, VEGF-dependent alterations, non-VEGF pathways, and stromal cell interactions [13–17]. Tumour cells may become accustomed to hypoxia or nutrient deprivation or induce angiogenesis via other growth factors [18, 19]. Such events can lead to higher survival levels of the tumour cells. Moreover, some antiangiogenic agents can cause side effects such as acne-like rash, hypertension, and diarrhoea [20, 21].

Traditional Chinese medicine is valuable for the treatment of various diseases, especially refractory diseases. More importantly, some studies proved that traditional Chinese medicine inhibits endothelial progenitor cells migration and tube formation without any cytotoxic activity [22]. Coadministration of traditional Chinese medicine and chemotherapy drugs could regulate angiogenesis, favour the delivery of chemotherapy drugs to the tumour lesion, promote apoptosis of tumour cells, enhance the effective treatment performance of chemotherapy drugs, and minimize the toxic side effects caused by chemotherapy drugs [23, 24]. Therefore, traditional Chinese medicine is more effective and safer in treating diseases. Previously, we found that many herbal extracts such as *Epimedium brevicornu* Maxim, *Dalbergia odorifera* T. Chen, and *Trichosanthes kirilowii* Maxim can regulate angiogenesis [25]. Cucurbitacin E, a compound in herbal extracts, can inhibit tumour angiogenesis by inhibiting vascular endothelial growth factor receptor 2- (KDR/VEGFR2-) mediated Jak-STAT3 and mitogen-activated protein kinase (MAPK) signalling pathways [26]. Astragaloside IV and curcumin can suppress the expression of fibroblast growth factor-2, matrix metalloproteinase 2, VEGF, hepatocyte growth factor, thrombosis-related factor tissue factor, and coagulation factor VII, thereby reducing the microvessel count [27]. The above herbal studies focused on single or several compounds related to angiogenesis. Nevertheless, during treatment, multiple herbal compounds interact or cross-react to regulate different targets and pathways. Thus, even though traditional Chinese medicine is safe for clinical treatment, the *in vivo* intervention mechanism is diverse, complex, and largely unknown.

**Figure 1:**
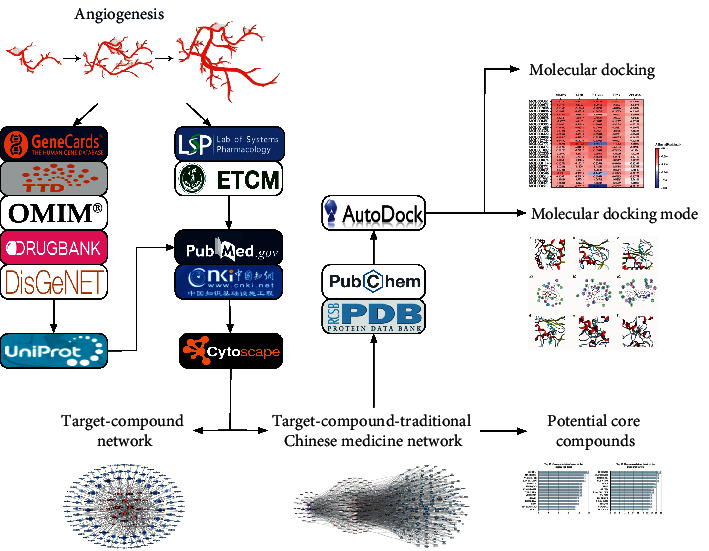
The workflow of the investigation.

Network pharmacology is a combination of pharmacology, biomedicine, systems biology, and network biology. Through various methods such as data mining, statistics, and modelling information visualization to recognize multicomponent synergy and assess each specific agent combinations' synergistic relationship [28–30], the mechanism of drugs in the biological network was analysed as well as determining which pathophysiological mechanisms are involved in the disease [28]. In this study, the angiogenesis targets were explored through network pharmacology, and the corresponding compounds and herbs were matched. Furthermore, the effect of herbal compounds on angiogenesis and the intervention mechanism was demonstrated. The findings of this study are expected to provide insights into the development of novel therapeutics for angiogenesis. The detailed workflow of the investigation is shown in Figure 1.

## 2. Materials and Methods

### 2.1. Collection of Data regarding Angiogenesis-Related Targets

Data were collected from GeneCards (https://www.genecards.org/) [31], Therapeutic Target Database (TTD; http://db.idrblab.net/ttd/) [32], Online Mendelian Inheritance in Man (OMIM, https://omim.org) [33], DrugBank (http://www.drugbank.ca) [34], and DisGeNET (http://www.disgenet.org) [35]. The keyword used to search these databases was “angiogenesis.” After sorting and removing repeated targets, matching targets' full name was established using the UniProt database (https://www.uniprot.org/) [36].

### 2.2. Screening of Related Herbal Compounds

The targets were used to match the compounds related to angiogenesis in the Traditional Chinese Medicine Systems Pharmacology Database and Analysis Platform (TCMSP; http://tcmspw.com/) [37] and Encyclopedia of Traditional Chinese Medicine (ETCM; http://www.tcmip.cn/ETCM/index.php/Home/Index/) [38]. The compounds and targets were imported to Cytoscape version 3.8.1 (Institute for Systems Biology, Seattle, WA, USA) [39] to construct a “target-compound” network. After preliminary screening, the related compounds were verified through a literature review, and the compounds closely related to diseases were screened as related compounds.

### 2.3. Collection of Related Traditional Chinese Medicines and Construction of a Target-Compound-Traditional Chinese Medicine Network

By collecting the traditional Chinese medicines related to compounds and constructing the “compound-traditional Chinese medicine” network combined with the “compound-target” network, the “target-compound-traditional Chinese medicine” network was constructed using Cytoscape 3.8.1 to explore and mine the relationships within the network. Key nodes were found by calculating the topological parameters of each node in the network using NetworkAnalyzer to preliminarily evaluate the effectiveness of traditional Chinese medicine and the compounds on angiogenesis.

### 2.4. Statistics and Frequency Analysis of Traditional Chinese Medicine Information

Information related to traditional Chinese medicine, including the four natures, five flavours, and meridians, was collected from the Chinese Pharmacopoeia (2020 edition) [40], the 13th five-year plan textbook of Traditional Chinese Medicine Pharmacy [41], and the Chinese Dictionary of Clinical Drugs [42]. If the information did not exist in these three documents, that medicine was excluded. IBM SPSS Statistics 26.0 was used to perform statistical tests and frequency analysis.

### 2.5. Molecular Docking for Targets and Compounds

To define the reliability of the interaction relationship between the core targets and core components in the “target-compound-traditional Chinese medicine network” and explore the new drug–target combinations, the top five targets with a moderate value for target-compound-traditional Chinese medicine were selected as receptors. The crystal structures of these proteins were selected and preserved in PDB format from Biological Macromolecular Structures Enabling Breakthroughs in Research and Education (RCSB; http://www.rcsb.org/pdb/). The 3D structures of the candidate compounds were downloaded and saved in SDF format from PubChem (https://pubchem.ncbi.nlm.nih.gov/). These SDF files were converted to the PDB format using Open Babel. The water molecules in the ligands were removed using AutoDock Tools 1.5.6 (Molecular Graphics Lab, La Jolla, CA, USA). After dispersing the ligands and receptors, nonpolar hydrogen bonds were added, and Gasteiger charges were calibrated and stored as pdbqt files. The selected potential core ligands were treated with energy minimisation, and the ligand atom type and calculated charge were saved in the pdbqt format. AutoDock Vina 1.1.2 [43] was used to calculate the docking score between the target and ligand to evaluate its matching degree and docking activity. A docking score of less than −4.25 indicated binding between the ligand and target. A score of less than −5.0 indicated better binding activity, and a score of less than −7.0 indicated vigorous docking activity. The ideal combinations were selected according to the affinity value, and the molecular docking pattern was displayed by MOE2019.

## 3. Results

### 3.1. Target Acquisition

We obtained 4609 targets related to angiogenesis from the GeneCards database. We identified the targets with strong correlation by calculating the median of their correlation coefficients because of the large number of targets. After six calculations, the medians were 0.65, 1.39, 3.25, 4.96, 7.47, and 10.52, and 74 targets with higher correlation coefficients were obtained. Furthermore, 5, 1, 0, and 0 related targets were separately obtained from the TTD, OMIM, DrugBank, and DisGeNET databases, respectively. After removing duplicate values and standardising them using the UniProt database, 79 angiogenesis targets were finally acquired. Containing the target information from TCMSP and ETCM databases were 49 and 4 target information, respectively. However, four were duplicate targets, and eight targets did not match any compound because the databases did not have information on related ingredients. Thus, only 41 targets were matched with small-molecule compounds and became potential targets.  Table 1 shows the targets with over ten corresponding compounds.

**Figure 2:**
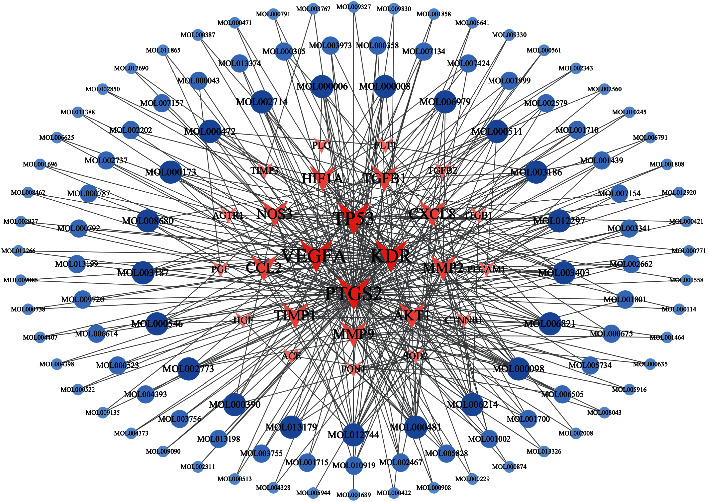
Target-compound network.

A total of 3839 small-molecule compounds were matched with 41 potential targets to construct a target-compound network consisting of 3440 nodes and 3839 edges. Although many small-molecule compounds showed a match, some were less related to the target or were associated with fewer studies. Hence, under the “Degree >1” condition, the targets and compounds with a greater degree of interaction were screened out, resulting in 28 targets and 264 candidate compounds. These were then screened through a literature review for *in vivo* and *in vitro* activities. Finally, 110 compounds with research significance and 26 related targets were determined; these were used to construct the “target-compound” network (Figure 2), which contained 136 nodes and 370 edges.

### 3.2. Identification of Traditional Chinese Medicines and Target-Compound-Chinese Medicine Network Construction

**Figure 3:**
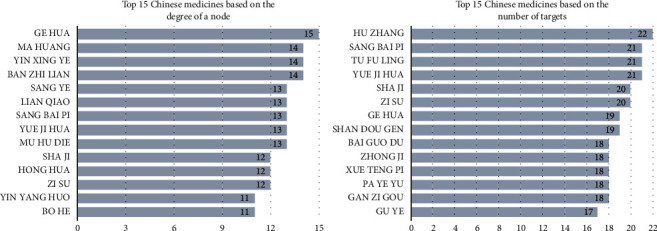
Number of candidate compounds and targets related to traditional Chinese medicines.

A total of 447 Chinese medicines were obtained from 110 candidate compounds through database and literature matching. A compound-Chinese medicine network was first constructed according to the relationship between the compounds and Chinese medicines, and it contained 594 nodes and 2240 edges. Based on the connections of the node, the top nine Chinese medicines were Puerariae Flos (Ge Hua), Ephedra Herba (Ma Huang), Ginkgo Folium (Yin Xing Ye), Scutellariae Barbatae Herba (Ban Zhi Lian), Mori Folium (Sang Ye), Forsythiae Fructus (Lian Qiao), *Morus alba* Root Bark (Sang Bai Pi), Rosae Chinensis Flos (Yue Ji Hua), and Oroxyli Semen (Mu Hu Die), which contain the candidate compounds 15, 14, 14, 14, 13, 13, 13, 13, and 13, respectively. Through the bridging effect of candidate compounds, the targets of various Chinese medicines were obtained. The top six Chinese medicines were Polygoni Cuspidati Rhizoma et Radix (Hu Zhang), *Morus alba* Root Bark (Sang Bai Pi), Smilacis Glabrae Rhizoma (Tu Fu Ling), Rosae Chinensis Flos (Yue Ji Hua), *Hippophae fructus* (Sha Ji), and *Perilla frutescens* (Zi Su), which contain compounds 22, 21, 21, 21, 20, and 20, respectively. Therefore, it can be inferred that these six Chinese medicines have a strong regulatory effect on the development of angiogenesis.  Figure 3 shows the top 15 candidate compounds of traditional Chinese medicines based on the number of related targets and the degree of a node in the compound-Chinese medicine network. According to a previous study, the median compound degree value of the network was seven [44]. According to the three conditions of closeness centrality, betweenness centrality, and compound degree value greater than 20, there were 27 potential core compounds. The top five ingredients were quercetin, *ββ*-sitosterol, kaempferol, luteolin, and ursolic acid. The remaining potential core compounds are shown in Table 2.

**Figure 4:**
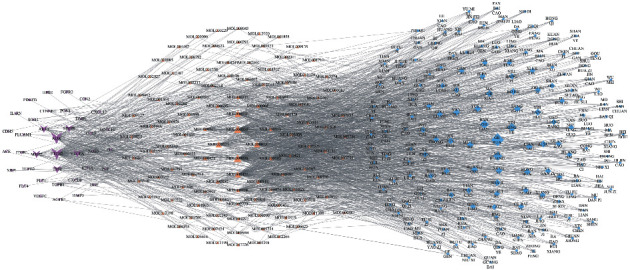
Target-compound-traditional Chinese medicine network. The diamond-shaped nodes represent Chinese medicine, the triangular nodes represent the ingredients, and the V-shaped nodes represent the target. The icon size of each node is positively correlated with its degree value.

The target-compound-traditional Chinese medicine network was reconstructed by selecting traditional Chinese medicines with a degree > 4. Their associated compounds and targets were organised to display the relationships among angiogenesis-related targets, compounds, and traditional Chinese medicines more intuitively (Figure 4).

### 3.3. Statistics and Frequency Analysis of Chinese Medicine Information

**Figure 5:**
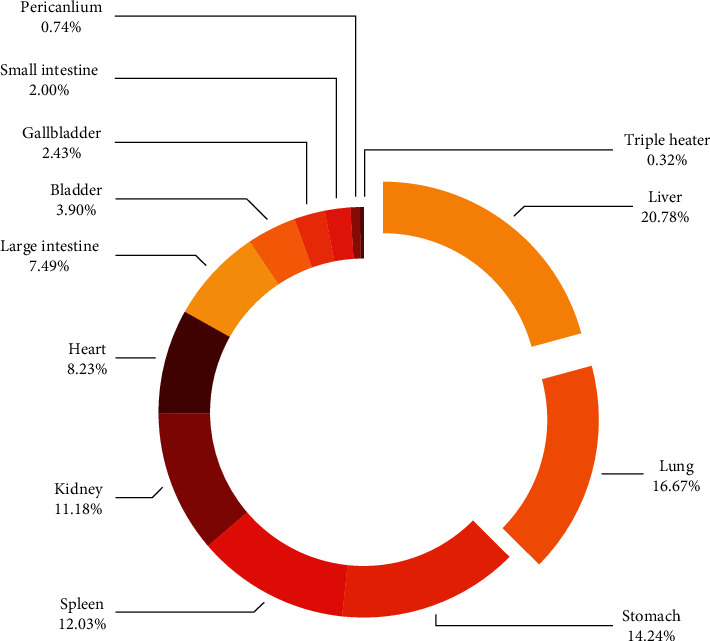
Channel entry of traditional Chinese medicine regulating angiogenesis.

Information regarding flavour, natures, and meridian was collected and analysed for 413 traditional Chinese medicines. The highest frequencies of the four natures were warm and cold, accounting for 25.5% and 24.5%, respectively. Flavour analysis revealed that the most predominant flavours were acrid and bitter, accounting for 28.9% and 27.6%, respectively. The highest-ranked meridians related to angiogenesis were the liver, lungs, and stomach, accounting for 20.8%, 16.7%, and 14.2%, respectively. The results of the statistical analyses are shown in Table 3 and Figure 5.

### 3.4. Molecular Docking

The 27 core potential compounds were molecularly docked with five core targets, namely, matrix metallopeptidase 9 (MMP9), VEGFR2, prostaglandin-endoperoxide synthase 2 (PTGS2), TP53, and vascular endothelial growth factor A (VEGFA), and 135 sets of receptor-ligand docking results were obtained. Among the 135 receptor-ligand groups, 94 groups (69.63%) showed affinity <−5, and 22 groups (16.30%) showed affinity <−7.

Among the 135 combinations, 55 combinations were present in the target-compound network. Among these 55 combinations, the highest score for docking was observed for PTGS2-astragalin (−9.18 kcal/mol), and the lowest docking score was observed for PTGS2-ursolic acid (4.20 kcal/mol). The average of the above combinations was −5.56 kcal/mol. This result indicates that the screened potential core compounds may have better binding activity with the core target and supports the reliability of drug-target interactions in the target-compound network to a certain extent.

**Figure 6:**
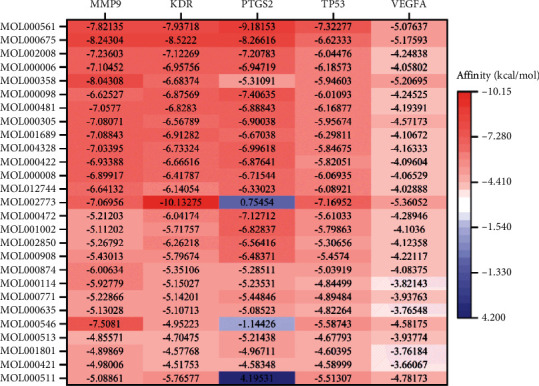
Molecular docking results.

Molecular docking results revealed 80 new combinations outside the target-compound network. The more ideal combinations outside the target-compound network were KDR-beta-carotene (-10.13 kcal/mol), MMP9-beta-sitosterol (−8.04 kcal/mol), MMP9-astragalin (−7.82 kcal/mol), and MMP9-diosgenin (−7.51 kcal/mol). There were 52 new combinations with affinity <−5 kcal/mol, suggesting that they all have good docking activity. The docking activity of these four combinations exceeded that of most combinations in the target-compound network; therefore, these are more likely to have a strong drug-target relationship. These docking results can provide data for the experimental screening and design of related Chinese medicines and ingredients in the future. The results are shown in Figure 6.

**Figure 7:**
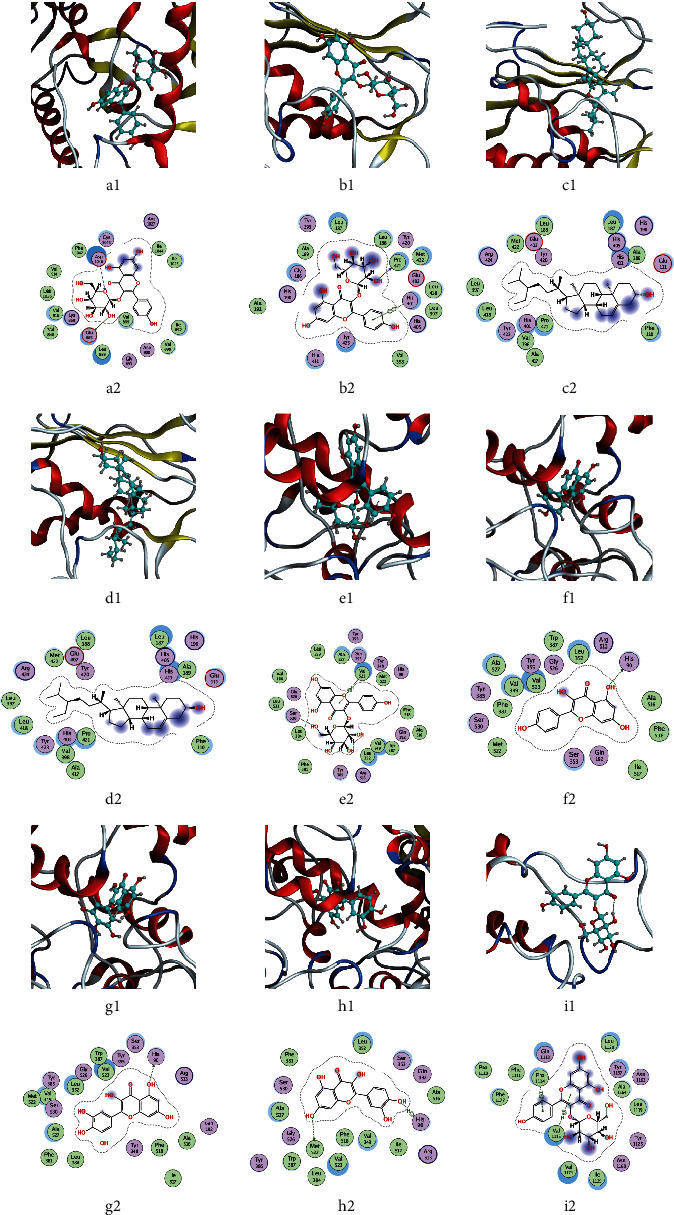
Molecular docking model. In the 3D structure of ligand-protein complexes, the protein backbone is represented as a cartoon (*α*-helices in red and *ββ*-sheets in green), peptide chains are coloured differently, and ligands are displayed in blue. The 2D interaction model shows amino acids circled differently according to their propensity for water; green: hydrophobic residues; purple: polar residues. (a) KDR-6gqq-astragalin; (b) MMP9-1gkc-astragalin; (c) MMP9-1gkc-beta-diosgenin; (d) MMP9-1gkc-beta-sitosterol; (e) PTGS2-5ikq-astragalin; (f) PTGS2-5ikq-kaempferol; (g) PTGS2-5ikq-myricetin; (h) PTGS2-5ikq-quercetin; (i) tp53-1jsp-astragalin.

Considering the ideal combination of the affinity value of molecular docking and degree value of the target-compound-drug network, nine more ideal combinations were selected. Their docking conditions are displayed in three-dimensional and two-dimensional molecular docking patterns. Each ligand was embedded in the active pocket of the target and interacted with multiple residues of the target through hydrophobic interaction and hydrogen bond formation (Figure 7).

## 4. Discussion

Angiogenesis is a complex process that requires the coordinated regulation of several activating and inhibitory pathways. It participates in developing many diseases, such as cancers, atherosclerosis, rheumatoid arthritis, hepatitis, and inflammation. There are many factors involved in the regulation of angiogenesis. As traditional Chinese medicines, which have a curative effect in clinical treatment, are applied in combination, they contain multiple components and targets. Therefore, elucidating the mechanism and exploring the potential components of traditional Chinese medicines are of great significance in developing novel drugs.

### 4.1. Target

According to the target-compound network screening results, the top scores were obtained for PTGS2, KDR, VEGFA, and MMP9. The primary role of PTGS2 in angiogenesis is to induce the synthesis of individual prostanoids such as PGD2, PGE2, PGF2a, PGI2, and TXA2. Prostaglandin (PG) can boost VEGF production in a paracrine, intracrine, or autocrine manner. Moreover, VEGF stimulates PTGS2 expression, thereby triggering PG production. This, in turn, increases the levels of PGs and stimulates the expression of angiogenic factors such as VEGF and bFGF [45]. VEGF expression is regulated by many factors such as epidermal growth factor, hypoxia-inducible factor (HIF), and platelet-derived growth factor (PDGF). During angiogenesis, VEGF signalling regulates the activities of several kinases through VEGFR2 and guides the proliferation, migration, and survival of cells.

An increased number of endothelial cells, both tip and stalk cells, is a significant feature of vascular proliferation. Endothelial tip cells are induced by VEGF gradients and promote the formation of filopodia. The molecular regulation of these events occurs via the activation of Notch signalling and increased expression of Notch ligands on endothelial cells. A high level of Notch signalling can decrease VEGFR2 expression. Physiological homeostasis requires this negative feedback loop [46]. One crucial event implicated in the migration and proliferation of vascular endothelial cells is the proteolytic degradation of basement membranes and extracellular matrix (ECM) components by matrix metalloproteinases (MMPs) [47]. The secretion of MMPs allows endothelial cells to penetrate their underlying basement membrane and eliminate the contact inhibition that blocks endothelial cell proliferation [48]. The gene expression of MMPs may stimulate the production and secretion of major proangiogenic factors such as VEGF and fibroblast growth factor-2, which promote angiogenesis [48–50]. MMP9 cleaves ECM proteins and activates cytokines and chemokines to regulate tissue remodelling [51]. In the intracardiac injection experiment, the injected ECM-derived substance promoted cell attachment, migration, and proliferation, induced extracellular signal-regulated kinase (ERK) 1/2 activation, and promoted arteriogenesis [50]. In summary, the above targets play an essential role in regulating angiogenesis, and they are the preferred targets for traditional Chinese medicine intervention in angiogenesis.

### 4.2. Ingredients

Molecular docking showed that the components that bind well to the targets are astragaloside, kaempferol, myricetin, quercetin, and *β*-sitosterol. Astragalin suppresses interleukin-1*β*- (IL-1*β*-) induced inflammatory mediators by activating peroxisome proliferator-activated receptor-*γ*, which subsequently inhibits IL-1*β*-induced nuclear factor- (NF-) *κ*B and MAPK activation [52]. NF-*κ*B subunit p65 activates the transcription of HIF-1*α* and its target gene VEGF-A. Regulating HIF-1*α* via NF-*κ*B activation can contribute to angiogenesis [53]. Kaempferol is an antioxidant that reduces reactive oxygen species (ROS) metabolism by inhibiting the NF-*κ*B pathway and upregulation of the associated transcriptional pathway [54]. ROS regulate angiogenesis via two different mechanisms: the HIF-VEGF/VEGFR2 signalling pathway and the VEGF-independent mechanism involving the generation of lipid oxidation products [55]. Endothelial nitric oxide synthase (eNOS) plays an essential role in regulating cell migration activities and vascular permeability [56]. Myricetin and quercetin inhibit thioredoxin reductase (TrxR) in an NADPH- and concentration-dependent manner [57]. TrxR is a part of the thioredoxin (Trx) system, including Trx and NADPH [58]. This system plays essential roles in regulating cellular redox signalling and contributes to the regulation of VEGF-mediated signalling [59–61]. For example, Trx1, in endothelial cells, prevents von Hippel-Lindau-mediated degradation of the transcription factor HIF1, leading to the induction of VEGF expression [62]. The action mechanism of myricetin and quercetin in angiogenesis is not yet well understood, but it may be related to the Trx system. *β*-sitosterol administration was reported to reduce the expression of chemokines and the activity of MMP2 and MMP9 [63]. In summary, the above components of traditional Chinese medicine can be investigated for intervention in angiogenesis.

### 4.3. Chinese Medicine

An experiment using umbilical vein endothelial cells demonstrated that angiogenesis could be regulated by the extract of Polygoni Cuspidati Rhizoma et Radix via the inhibition of the phosphorylation of downstream signalling molecules such as ERK, Akt, and eNOS by VEGF/VEGFR2 [64]. These molecules can regulate endothelial cell survival, proliferation, and migration [65, 66]. The extract of *Morus alba* Root Bark inhibits the proliferation and migration of vascular smooth muscle cells induced by PDGF. It stimulates the formation of nitric oxide (NO) in endothelial cells [67]. NO is a vital gaseous signalling molecule that participates in the growth and remodelling of essential biochemical and molecular reactions necessary for regulating angiogenesis. The NO-induced activation of soluble guanylate cyclase increases cyclic guanosine monophosphate formation and protein kinase G activity to modulate signalling cascades by phosphorylation MAPKs, which successively phosphorylate and activate downstream proteins such as ERK1/2 [68,69]. These events regulate the proliferation and migration of endothelial cells, resulting in angiogenesis. Forsythiae Fructus aqueous extract triggers the inhibition of oxidative stress and inflammation via the MAPKs/Nrf2/HO-1 signalling pathway and inhibits cancer cell proliferation and angiogenesis [70].

Traditional Chinese medicines can be classified based on the four natures, five flavours, and meridians. Information about these factors can be related to efficacy and utility in a clinical setting [71]. In this study, the most prevalent natures were found to be warm and cold. According to Chapter 74 of the Zhizhenyao Dalunpian: Discussion on the Most Important and Abstruse Theory - Plain Questions, which describes the rules of using traditional Chinese medicine in treatment, diseases caused by cold are treated with warm-natured therapy, and heat syndromes are treated with cold-natured therapy.

The core function of warm is to activate blood, replenish qi, and prevent the inhibition of water. It is usually used in treating yang deficiency or problems in fluid transformation such as heart failure. In some studies, it is shown that the extract of the warm-natured drugs such as Allicin [72] and astragaloside IV [73], through regulating sarcoplasmic reticulum Ca2+ pump, improved the distribution and expression of PECAM-1, enhanced the migration and angiogenesis ability of cardiac microvascular endothelial cells, promoted angiogenesis, protected myocardial ischemia, inhibited cardiac hypertrophy and fibrosis, and reduced myocardial cell apoptosis. The primary function of cold is heat-clearing, detoxicating, and draining fire. It inhibited the release of inflammatory factors to treat the diseases caused by inflammation [74] such as cancer. Some extracts of the cold-natured drugs such as tetrandrine [75], artemisinin [76], and andrographolide [77] inhibited endothelial cell proliferation, adhesion, invasion, and tube formation by targeting vascular endothelial growth factor and blocking angiogenesis and invasion of cancer cells

Moreover, the highest-ranked flavours were acrid, bitter, and sweet. Acrid promotes diffusion and outthrust with dissipation—it is favourable for treating blood-stasis, blockage, and accumulations, such as ischemic stroke and cancer. Galangin and tetramethylpyrazine were extracted and isolated from traditional Chinese medicine with acrid flavours. Galangin ameliorated neurological scores, cerebral infarct volume, and cerebral edema through improving the neurovascular microenvironment [78]. Tetramethylpyrazine restrains angiogenesis by suppressing the ERK1/2 and Akt pathways and promotes apoptosis of tumour cells [24].

Furthermore, the bitter flavour and cold-natured drugs exerted a similar effect on draining fire, but the bitterness was better to dry dampness and drain fire downward. It also has an excellent therapeutic effect on diseases caused by inflammation. The primary function of sweet is to supplement and harmonize the centre and relax tension. It is appropriate for patients who are in pain and asthenia. Under the Chinese medicine theory, diabetic retinopathy was often classified as the pattern of dual vacuity of qi and yin and was influential in treating diabetic retinopathy by boosting qi and nourishing, clearing heat, and breeding body fluids [79]. Plantaginis semen belongs to the extract of sweet medicinals, which ameliorated diabetic retinopathy through increasing vascular permeability and retinal vessel diameter and restrained retinal vascular dilation [80].

The theory of channel tropism posits that medicines have selective therapeutic effects in different Zang-fu organs. This study shows that the top two meridians related to the studied traditional medicines were the liver and lungs. The liver is a crucial organ that regulates blood, including its storage, filtration, and bleeding. In contrast, the lungs control the qi and are intimately related to the qi of the chest. They also play an essential role in the blood and qi movement in circulation in the body. Thus, the liver and lungs closely collaborate in regulating the qi and blood. Because of the above, it can be presumed that impaired angiogenesis, according to the basic theory of Chinese medicine, is associated with the failure of the liver and lungs to perform their functions. Qi stagnation and blood stasis cause poor circulation of blood, which leads to the collection of water-humour, with the stagnant qi being transformed into heat. Therefore, traditional Chinese medicine's fundamental treatment principles are to warm the yang, rectify the qi, and activate the blood. In cases of long-time ailment, efforts should be made to eliminate dampness and clear heat.

## 5. Conclusions

In this study, we used network pharmacology to identify proteins related to angiogenesis through databases and documentation. Also, we constructed a target-compound-traditional Chinese medicine network, which was explored and analysed for the potential compounds and mechanisms of traditional Chinese medicine that participated in angiogenesis. The findings of this study can effectively narrow the scope of screening, improve scientific research efficiency, and reduce economic costs in the research of therapeutic agents to treat angiogenesis. However, this study is preliminary and is based on database analysis; therefore, it does not fully demonstrate the actual situation or verify the participation of traditional Chinese medicine in angiogenesis *in vivo*. The specific molecular mechanism still needs to be explored through subsequent experimental research.

## Figures and Tables

**Table 1:** Target's information.

Number	Gene	UniProt number	Protein
1	PTGS2	P35354	Prostaglandin G/H synthase 2
2	KDR	P35968	Vascular endothelial growth factor receptor 2
3	VEGFA	P15692	Vascular endothelial growth factor A
4	FGF1	P05230	Acidic fibroblast growth factor
5	TP53	P04637	Cellular tumour antigen p53
6	MMP9	P14780	Matrix metalloproteinase-9
7	AKT1	P31749	RAC-alpha serine/threonine-protein kinase
8	MMP2	P08253	72 kDa type IV collagenase
9	CXCL8	P10145	Interleukin-8
10	FGF2	P09038	Basic fibroblast growth factor
11	HIF1A	Q16665	Hypoxia-inducible factor 1-alpha
12	TGFB1	P01137	Transforming growth factor beta-1
13	CCL2	P13500	C-C motif chemokine 2
14	NOS3	P29474	Nitric oxide synthase, endothelial

Identification of candidate compounds and target-compound network construction.

**Table 2:** Candidate compounds and targets information (degree > 20).

MolID	CAS	MolName	Degree	OB	DL
MOL000098	117-39-5	Quercetin	201	46.43	0.28
MOL000358	83-46-5	Beta-sitosterol	192	36.91	0.75
MOL000422	520-18-3	Kaempferol	136	41.88	0.24
MOL000675	112-80-1	Oleic acid	120	33.13	0.14
MOL000006	491-70-3	Luteolin	99	36.16	0.25
MOL000511	77-52-1	Ursolic acid	88	16.77	0.75
MOL000008	520-36-5	Apigenin	87	23.06	0.21
MOL000114	121-34-6	Vanillic acid	86	35.47	0.04
MOL000305	143-07-7	Lauric acid	69	23.59	0.04
MOL000513	149-91-7	3,4,5-Trihydroxybenzoic acid (Gallic acid)	57	31.69	0.04
MOL000908	515-13-9	Beta-elemene	51	25.63	0.06
MOL000771	501-98-4	p-Coumaric acid	48	43.29	0.04
MOL000635	121-33-5	Vanillin	48	52.00	0.03
MOL000561	480-10-4	Astragalin	39	14.03	0.74
MOL000472	518-82-1	Emodin	37	24.40	0.24
MOL002850	128-37-0	Butylated hydroxytoluene	35	40.0	0.07
MOL002773	7235-40-7	Beta-carotene	34	37.18	0.58
MOL001801	69-72-7	Salicylic acid	30	32.13	0.03
MOL000874	552-41-0	Paeonol	30	28.79	0.04
MOL000481	446-72-0	Genistein	26	17.93	0.21
MOL000421	59-67-6	Nicotinic acid	25	47.65	0.02
MOL002008	529-44-2	Myricetin	25	13.75	0.31
MOL004328	67604-48-2	Naringenin	24	59.29	0.21
MOL001689	480-44-4	Acacetin	23	34.97	0.24
MOL012744	501-36-0	Resveratrol	23	19.07	0.11
MOL001002	476-66-4	Ellagic acid	21	43.06	0.43
MOL000546	512-04-9	Diosgenin	20	80.88	0.81

**Table 3:** Natures and flavours of traditional Chinese medicine regulating angiogenesis.

Four natures	Frequency	Percentage	Five flavours	Frequency	Percentage
Warm	103	25.5	Acrid	160	28.9
Cold	99	24.5	Bitter	153	27.6
Neutral	84	20.8	Sweet	139	25.1
Slightly cold	49	12.1	Sour	27	4.9
Cool	34	8.4	Astringent	25	4.5
Slightly warm	23	5.7	Slightly bitter	22	4
Hot	10	2.5	Salty	12	2.2
Great cold	1	0.2	Bland	9	1.6
Great hot	1	0.2	Slightly acrid	3	0.5
			Slightly sweet	3	0.5
			Slightly sour	1	0.2

## Data Availability

The data and materials used for this study will be made available by the corresponding author Qingyong He upon reasonable request.

## Abbreviations

ECM: Extracellular matrix
ETCM: Encyclopedia of Traditional Chinese Medicine
HIF: Hypoxia-inducible factor
OMIM: Online Mendelian Inheritance in Man
PDGF: Platelet-derived growth factor
ROS: Reactive oxygen species
TTD: Therapeutic Target Database
VEGF: Vascular endothelial cell growth factor.
